# Intensity of Oestrus Signalling Is the Most Relevant Indicator for Animal Well-Being in High-Producing Dairy Cows

**DOI:** 10.4061/2011/540830

**Published:** 2011-08-22

**Authors:** Emanuel Garcia, Jan Hultgren, Pontus Fällman, Johanna Geust, Bo Algers, George Stilwell, Stefan Gunnarsson, Heriberto Rodriguez-Martinez

**Affiliations:** ^1^Department of Animal Environment and Health, Swedish University of Agricultural Sciences, 532 23 Skara, Sweden; ^2^Nötcenter Viken, Vikens Egendom, 521 91 Falköping, Sweden; ^3^Centro de Investigação Interdisciplinar em Sanidade Animal, Universidade Técnica de Lisboa, 1300-477 Lisboa, Portugal; ^4^Department of Clinical and Experimental Medicine, Faculty of Health Sciences, Linköping University, 581 85 Linköping, Sweden

## Abstract

Full signalling of oestrous behaviour is vital for proper timing of AI and good reproductive performance, currently jeopardized by shorter observations of oestrus behaviour. Alternative indicators including progesterone (P_4_) recordings on-farm are tested. Oestrous intensity of 37 heifers (H) and 30 1st-parity dairy cows (C_1_) either Swedish Red (32) or Swedish Holstein (35) with high genetic potential for milk production, was studied in relation to AI. P_4_-levels in blood or milk were monitored on-farm at 0, 7, and 20 d post-AI with a portable ELISA reader (eProCheck^800^). Avoidance distance and body condition were scored at day 7, and pregnancy diagnosed by P_4_ (day 20) and trans-rectal palpation (day 50). More heifers (46%) than C_1_-cows (10%) showed standing oestrus (strongest intensity, *P* < 0.05), leading to higher pregnancy rate at d50 (72% versus 37% for C1, *P* < 0.01) and calving rate (H: 64%, C1: 33%, *P* < 0.05). Avoidance distances were short (<1 m), reflecting good human-animal interaction. Visually-recorded standing oestrus yielded 4.8 fold higher odds of pregnancy, respectively 4.6-fold higher odds of calving. On-farm P4-recordings had complementary value yet less accuracy. Intensity of oestrus signalling relates to animal well-being, reflected in pregnancy-to-term being a good indicator for optimal welfare in high-producing dairy cattle.

## 1. Introduction

Between 1997 and 2007, world milk production increased by 27% (122 million tonnes) [1]. By 2009, prevailing input costs, alongside the complex worldwide recession, led to a dramatic fall in milk prices [2] and a subsequent decrease in the rate of expected milk production growth [3]. Milk volumes were maintained by a gradually reduced number of high-producing cows. However, high milk production can negatively affect animal welfare [4, 5], including the documented global decrease in average dairy herd reproductive performance, mainly in the dominating American Holstein breed. The latter is a backlash of the genetic global use of a limited base of artificial insemination (AI) sires, which, being mainly selected for milk yield but not for health or reproductive traits, have decreased cow robustness in this highly industrialized sector of animal production [6].

Many reproductive disorders reduce, via stress or painful inflammatory reactions, animal welfare at short or long term [4]. In modern dairy farming, signs of standing oestrus are not always displayed or recorded, and they are considered decreasing figures, owing to the concerted action of many factors, primarily due to the inability of the cow to freely display oestrus signs as a consequence of metabolic stress and energy imbalance due to high milk yield and other pathologies (mastitis, lameness, etc.) as well as personnel scarcity [7–9]. Yet neither expression of oestrus nor reproductive performance have been listed as criteria for on-farm welfare assessment [10]. In a large survey performed in Sweden, reproductive performance was, however, ranked as a good indicator for animal welfare status in dairy herds [11]. This apparent variation among studies might relate to different conceptual interpretations of the relevance of oestrus signalling for animal well-being and the outcome of breeding. 

It is our working hypothesis that optimal behavioural display of oestrus indicates the level of well-being among dairy cattle [12]. Moreover, since full oestrous signals display female receptivity for mating in direct relation to spontaneous ovulation, oestrous intensity would facilitate proper timing for artificial insemination (AI) and, ultimately, affect reproductive performance. Although visual recording is by far the most accurate method to determine presence and intensity of oestrous signs, the restructuring of the dairy sector, with fewer caretakers per number of cattle, has most likely jeopardized proper oestrus detection, leading to improperly timed AI. Complementary methods, such as activity recorders and sophisticated on-line measuring of progesterone (P_4_) levels (reviewed by Rodriguez-Martinez et al. [6]), are being increasingly used instead, particularly in large herds. Whether they can compensate the lack of personnel to visually record oestrous events remains to be determined. 

Therefore, we have studied the value of direct observational assessment of oestrous intensity versus relative levels of P_4_ measured on-farm. We have also determined the relationships between intensity of oestrous signalling with well being indicators (avoidance distance scoring) and reproductive performance (as pregnancy and calving rates). The overall goal was to determine the value of oestrus intensity as a marker for good animal welfare in dairy heifers and first-parity cows with a high genetic potential for milk production.

## 2. Material and Methods

### 2.1. Ethical Permission

The study was approved in advance by the local Ethics Committee on Animal Research (Dnr 123-2008, Gothenburg, Sweden), assuring compliance with EC Directive 86/609/EEC for animal experiments.

### 2.2. Farm Description: Animals

The study was carried out at the dairy farm Nötcenter Viken (NCV) in Falköping, Sweden. The cattle herd consisted of ~1,000 heads, including ~400 milking cows of various parities and ~200 heifers. Cows were kept in two different parks, separated by breed, whereas heifers were distributed in three contiguous parks in another building. No bulls were present on the farm. The loose housing system at NCV consisted of cubicles equipped with rubber mattresses and concrete-based walkways coated with rubber-mats (cows) or not (heifers). The animals were in-house, except for the compulsory summer grazing period from May to September. Milking took place three times per day (0500 h, 1300 h, 2000 h) in a rotary milking parlour (DeLaval, Stockholm, Sweden). A total of 20 employees, working on shift basis, were responsible for managing the animals, including veterinary procedures and the MOET scheme. Animals were fed according to feeding recommendations from Lantmännen (LFU system, 1998 [13]). Basically, rations were composed of grass silage, cereal grains, sugar beet byproducts, and heat-treated rapeseed or soybean meal [13]. Minimum age for first AI was 15 months, and the voluntary waiting period after calving was 50 days. Heat detection was done routinely by NCV AI technicians, by a preliminary recording of activity with the software ALPRO (DeLaval, Stockholm, Sweden, www.delaval.com), followed by a single 1-hour long morning tour within all animal groups, including those in the milking parlour. The study included a total of 37 heifers (H) and 30 first-parity cows (C_1_ cows) of either SR (19 heifers, 13 C1-cows) or SH (18 heifers, 17 C1-cows) breeds, varying in body condition (BC), time from parturition, and number of previous AIs.

### 2.3. Study Design

The study was performed between 13 March and 27 May, 2009. Within the period of study, females identified by the NCV personnel (four AI technicians) to be in oestrus were selected for AI and included in the study, each animal contributing with only one record of AI and a possible pregnancy; that is, animals repeating oestrus did not reenter the study. The day of AI was defined as day 0 (d0). The semen used was from progeny-tested sires, selected based on their genetic background and breeding goals (VikingGenetics, Skara, Sweden), using a standard protocol for thawing (in water at 35°C for 12 seconds), and AI (semen deposited in the uterine body). The AI was done on the same day oestrus was detected, up to 1400 h. Milk production records were retrieved until 2 June 2009 to relate average daily milk yield to other variables.

On d0, signs of oestrus were further visually explored by the same independent observer (not NCV personnel) for about an extra morning hour, scoring for presence of mucous vulvar discharge, flehmen reflex, restlessness, licking or sniffing of the perineal region, butting, chin resting, mounted but not standing, mounting other cows (or attempt), and of standing heat. All signs were registered as 0 (absence) or 1 (presence). Restlessness was considered present when the cow showed high activity (ALPRO software recordings) or at visual observation. Signs of oestrus were scored (adapted from Van Eerdenburg et al. [7]) as being of *Low* intensity (not being mounted and not standing), *Medium* intensity (being mounted but not standing) or *High* intensity (standing steadily when mounted). Oestrous scores (OI), complemented with information from the AI technicians regarding ease of AI, and visible metoestrus bleeding, were registered as binary data. Peripheral blood was collected by tail venipuncture after visual assessment using 10 mL Venosafe vacuum tubes (Venosafe Clot Activator tubes and Venosafe heparin, Vacutainer, Mich, USA), for analysis of the relative contents of progesterone (P_4_) in serum and plasma. Additionally, milk was always collected before blood sampling from cows, also for P_4_ analysis.

On day 7 (d7), the inseminated animals were assessed for body condition (BC) and avoidance distance (AD), and blood and milk were once more collected and processed, as described above. BC scoring used the 5-point scale (with 0.5 increments) of the Swedish Dairy Association (adapted from Edmonson et al. [14]), and scores were revised using digital images taken from the rear of the cow at a 0 to 20° angle relative to the tail head as described by Ferguson et al. [15]. The AD was assessed before blood sampling, always by the same person. Each animal was tested twice, at the feeding rack and, immediately thereafter, inside the stall. While feeding, the animals were approached slowly frontwise at one step per second by the observer, one arm overhand in an angle of about 45° in front of the body, targeting the muzzle, until the cow withdrew or until touching. The distance between the cow's head and the hand was estimated at the moment of withdrawal [16]. In the stall, standing animals were approached from the front and avoidance estimated, as while feeding the proportions of animals that tolerated touch as well as the proportion of animals that allowed touch for more than 3 sec were recorded. 

All inseminated animals were visually checked for nonreturn to oestrus in the interval day 18 to day 23. On day 20 (d20), blood and milk were collected and processed, as described above for the inseminated animals. Those animals not returning to oestrus were checked for pregnancy by transrectal palpation on day 50. Events were recorded during pregnancy until partus.

### 2.4. Progesterone (P_4_) Levels in Peripheral Blood Serum/Plasma and Milk

Blood serum was collected after clotting and centrifugation at 300 × g for 10 min. Blood plasma was collected after centrifugation. Both serum and plasma were transferred to plastic tubes and either analysed immediately after harvesting (serum *n* = 94, plasma *n* = 86) or frozen to −20°C (serum *n* = 93, plasma *n* = 80) and analysed after thawing at room temperature. Milk, collected into 5 mL plastic tubes (*n* = 70), was maintained at room temperature for immediate analysis or refrigerated (+4°C) if the analysis was delayed for more than 15 min. Milk samples were analysed within 4 h of sampling following manual shacking (10 times).

An enzyme-linked immunosorbent assay (ELISA) test, run in the eProCheck^800^ processor (Minitüb, Tiefenbach, Germany [17]) was used to determine relative P_4_ levels in blood serum, blood plasma, or milk. Samples measured were 15 **μ**L for serum or plasma or 20–30 **μ**L for milk, allowing 1 to 7 samples to be tested at a time within a 20-minute analysis. Once loaded to the sample vial of the reader, the vial holder was inserted into the device, and the following procedures were automatically conducted: addition of conjugate, incubation, wash, addition of enzyme-substrate complex, incubation, and results output. P_4_ concentration was evaluated in the device by colour change, and from 1 to 7 samples could be tested at a time within a 20-minute analysis. The results of relative P_4_ levels were given by the processor as a bar graph with 6 levels (1–6), which, according to the manufacturer [17], corresponded to the following P_4_ concentration ranges (in nM/L): **1**: 0–1.3, **2**: 1.4–3.0, **3**: 3.1–4.5, **4**: 4.6–7.5, **5**: 7.6–9.9, **6**: >10, and which could be grouped as *Low* (levels 1-2, <3.0 nM/L), *Medium* (levels 3-4, 3.1–7.5 nM/L) or *High* (levels 5-6, >7.6 nM/L).

### 2.5. Statistical Analysis

The data were analysed using the SAS statistical package, version 9.1 for Windows (SAS Institute Inc., Cary, NC, USA). Spearman rank correlation analysis was used to determine relationships between variables. Differences in mean values and proportions were, respectively, examined with *t*-test (age, BCS, AD) and Fisher's exact test (category, breed, OI, lameness, pregnancy and calving rates) while the Kruskal-Wallis test was used for pair-wise comparisons. Analysis of variance (ANOVA) using the General Linear Models procedure with Tukey adjustment was used for comparison of avoidance distance in different OIs and P_4_ on day 7. Odd ratios were derived from a contingency table. Differences of *P* < 0.05 were considered statistically significant.

## 3. Results

The studied population (37 heifers and 30 C_1_-cows) was rather evenly distributed by breed (Heifers: SH-49% and SR-51%, C_1_-cows: SH-57% and SR-43%), with clear age differences (Heifers: 18 ± 2.2 (means ± SD, range 15–24) compared to C_1_ cows: 33.5 ± 7.0 (27–55), *P* < 0.01). Prevalence of BCS differed significantly (*P* < 0.05) between categories (Heifers: 3.4 ± 0.4 (points, range 2.5–4), C_1_-cows: 2.8 ± 0.4b (2–3.5)). Average milk production per day for the C_1_cows within the study period was 33.4 ± 5.2 kg (means ± SD).

Intra- and interassay comparisons were done (see Table 1) with CVs being below 5%, particularly when the relative P_4_ levels in the samples were extreme (either *High* [5, 6] or *Low* [1, 2]). Comparison of paired samples of various sources (plasma, serum, or milk) with the same relative P_4_ level (*Low*,*   Medium*, or*  High*) showed that serum versus plasma had the highest degree of similarity (73.2% of samples had the same P_4_ level, *n* = 224, 95% CI = 67.9–78.1%), followed by plasma versus milk (65.4% same P_4_-level, *n* = 81, 95% CI = 55.8–74.2%), and lastly, serum versus milk (55.6% same P_4_-level, *n* = 81, 95% CI = 45.8–65.0%). The most consistent sample volumes were 15 **μ**L for blood plasma or serum (either fresh or frozen) and 20 **μ**L for milk. 

*Low*, *Medium,* or *High* oestrous intensity (OI) was shown by 33 (49%), 14 (21%), or 20 (30%) of the 67 animals studied (heifers and C_1_-cows) at d0. Oestrous intensity was more often scored as *High* among heifers (17/37) than among C_1_-cows (3/30) (*P* < 0.01). For *Low* oestrous signs, however, there were no differences between animal categories (NS, *P* > 0.05). When observations were stratified by OI, animal category (heifers or C_1_-cows), and breed (SR or SH), there were no significant differences in relative P_4_ levels in blood serum at d0 (not significant, NS) (Table 2). Furthermore, there were no differences when comparing extreme oestrous intensity levels, that is, *Low* versus *High*, regarding proportions of the three P_4_ levels (not NS), indicating that oestrous intensity and relative P_4_ levels in peripheral blood were unrelated (Table 2 **)**. The P_4_ levels in peripheral blood varied widely. Relative P_4_ serum level at day 0 was *Low* in 47% of all studied animals (31/67 animals, 95%  CI = 65–83%), while 31% (20/67 animals, 95% CI = 22–41%) were *Medium* and 24% (16/67 animals, 95% CI = 16–34%) were *High*. Comparing categories of animals, only C_1_-cows showed a trend in the relationship between oestrous intensity and P_4_ levels, albeit being nonsignificant (NS), irrespective of breed. The distribution of animals having *Low* P_4_ levels at d0 did not vary significantly (NS) among the four oestrus-detecting technicians.

The avoidance distance tested either at the feeding rack or the stall yielded different distances in C_1_-cows than in heifers (30 versus 47 cm or 83 versus 103 cm, resp., NS), without a clear breed effect. There was a reasonable correlation between avoidance distances at the feeding rack and installs (*r* = 0.45, *P* < 0.001). The avoidance distance means were apparently shorter, although nonsignificant (NS), in animals having depicted *High* oestrous intensity. At the feeding rack, up to 35% (22/62) of the animals tolerated touch, of which 9 (15%) were heifers and 13 (21%) were C1-cows. Up to 27% (6/22) of animals could be touched for more than 3 seconds, 22% (2/9) of heifers and 31% (4/13) of cows. Finally, 11% of the C_1_-cows (3/27) tolerated being touched in the stalls, but none of them for more than 3 seconds. Cow age correlated positively with avoidance distance at the feeding rack (*r* = 0.57). Comparing the two locations for avoidance distance assessment, there was a moderate positive correlation (*r* = 0.49, *P* < 0.05), confirming to some degree the concordance of the procedures. One heifer had to be euthanised due to severe lesions after an accident in the stall and the data were removed from further analyses.

Overall pregnancy rate at d50 was 56% (37/66 animals), while overall calving rate reached 50% (33/66 animals). Up to 80% of the animals depicting *High* oestrous intensity became pregnant, compared to 45% depicting *Low* oestrous intensity at d0 (*P* < 0.05), whereas 46% of those depicting *Medium* intensity did not differ significantly from *High* or *Low*. Higher oestrous intensity related significantly to higher pregnancy (*r* = 0.28, *P* < 0.05). As expected, heifers had a higher pregnancy rate than C1-cows, 72% versus 37% (*P* < 0.01, Table 3). This difference was maintained for calving rates (64% versus 33% (*P* < 0.05, Table 3). Standing oestrus was associated with 4.8- and 4.6-fold higher odds of pregnancy calving, respectively. Pregnancy rate was highest among heifers with a low P_4_ level at d0 (80%), but it was considerably lower in corresponding C1-cows (31%) (*P* < 0.01, Table 3). Calving rates maintained the trends shown by pregnancy rate at d50 (Table 3). As expected, there was a strong correlation between P_4_ level at d20 and pregnancy rate (*r* = 0.55, *P* < 0.0001). The odds of females that showed standing oestrus (*High*) becoming pregnant, respectively, calving were 4.8- and 4.6-fold, respectively, higher (95%  CI = 1.38–16.45 resp., 95%  CI = 1.44–15.08) than for females showing only secondary oestrous signs (*Low-Medium*). Some animals became pregnant and subsequently calved despite a recorded high P_4_ level at d0 (6/11 heifers and 3/6 C1-cows). Considering C_1_-cows, the older the animal, the lower the oestrous intensity (*r* = −0.42, *P* < 0.05).

## 4. Discussion

The present study determined the presence of associations between intensity of oestrus signs and P_4_ levels in peripheral blood or milk with pregnancy-to-term outcome after AI and behavioural indices (avoidance distance testing), in Swedish Holstein or Swedish Red heifers and first-parity (C1) cows with high genetic potential for milk yield in a single nucleus herd having the same management and feeding routines. Overall pregnancy and calving rates (56% and 50%, resp.) were acceptable, with heifers having higher rates than C1 cows. Highest oestrous signalling was reflected in higher pregnancy/calving rates, standing oestrus having 4.8-to-4.6-fold higher odds of pregnancy calving, respectively, compared to detection based on secondary oestrus signs. Avoidance distance means (at either the feeding rack or the stall) were generally short (<1 m), but without significant relationship with oestrous intensity.

As expected, C1 cows depicted a lower OI display than heifers, which could be due to differences in housing, management, or incidence of lameness [9]. Further, the higher the intensity of oestrus, the higher the pregnancy and calving rates achieved (80%, and 75%, resp.). This finding is in agreement with Van Eerdenburg et al. [7], who found a shorter interval to ovulation (<24 h) in cows that scored (almost three times) higher intensity of oestrus. In the present study, pregnant animals scored higher than nonpregnant ones, clearly indicating that standing oestrus (as a token for full physiological signalling display) is still the best behavioural marker for successful AI. There is a trend in current dairy production for a decreased expression of oestrous signs [6]. This, together with increasing numbers of larger herds but with less personnel, led to an increased use of secondary signs, without standing heat or even hormonal measurements (as P_4_) to help decide when AI is to be performed. Oestrus of lower intensity is associated with delayed ovulation, reduced preovulatory oestradiol (E_2_) concentration, and poorer oocyte quality [18]. Obviously, it is of utmost importance to determine what causes low oestrous display.

The eProCheck^800^ automated ELISA reader primarily appeared to complement usual on-farm reproductive management, being able to determine P_4_ relative levels in most samples analysed either fresh (milk, serum, plasma) or frozen-thawed (serum, plasma). The highest degree of accuracy was for extreme values, for example, *Low* (<3.1 nM/L) or *High* (>7.6 mM/L) P_4_ concentrations, but variability was highest when milk was tested, suggesting that the instrument best analysed serum or plasma samples. However, for practical reasons, milk samples are easiest to retrieve under commercial conditions, and, thus, the instrument should preferably be adapted to this type of samples.

The decisions to breed were based solely on routine oestrus determinations by the personnel at Nötcenter Viken (NCV). Interestingly, if decisions had instead been based on P_4_ serum levels at d0, only 46% of the animals ought to have been inseminated; 59% if based on blood plasma and 90% if based on milk. However, nine animals with high P_4_ levels in blood serum became pregnant and calved, owing to either inaccurate readings or wrongly identified samples. Despite this flaw, the overall pregnancy rate in the animals studied did not differ substantially from what would have been obtained if breeding decisions had been based solely on eProCheck^800^ serum readings (55%), also when looking separately at heifers (72% versus 80%) and C_1_-cows (31% versus 37%). However, the number of inseminated animals would have been rather low, which makes us refrain from stronger conclusions. Considering a relative P_4_ level of >10 nM/L in serum, plasma, or milk as the true value on d20 in a pregnant cow resulted in a sensitivity estimate of the eProCheck^800^ of 97%, 92%, and 89%, respectively, and a specificity of 50%, 60%, and 75%, respectively. An alternative measure of accuracy was defined from a practical perspective, considering an animal as truly nonpregnant only if having a higher P_4_ level on d7 than on d20. This approach generated corresponding specificity estimates of 40%, 57%, and 46%, respectively. The eProCheck^800^ will hopefully be continuously upgraded to improve its accuracy.

The complete behavioural repertoire of oestrous display was observed in the studied animal population, except for the flehmen reflex (seen in other animals in the herd), and so the plurality of oestrous expression did not seem to be negatively affected. Oestrous intensity was classified as *Low* (slight signs) in almost half of the studied sample, while the other half accounted for *Medium* (21% of animals were mounted but not standing; mounting) or *High* (30% of animals showed standing oestrus) oestrous intensities, respectively. At first sight, this low display of strong oestrous intensity might result in a threat to fertility, especially if detection of oestrus is not optimized. Since the van Eerdenburg et al. [7] scoring system used here, albeit with slight modifications, clearly evidenced differences between heifers and C_1_ cows, it would be advisable to include it as routine at NCV. Oestrous intensity was lower in C_1_-cows compared to heifers, confirming the negative influence that high milk yield imposes, particularly among first calvers [6]. Although negative correlations were found between oestrous intensity variables and milk production in the present study, analysis of the whole lactation would be needed to draw more accurate conclusions. Moreover, the low variability in average milk production per day for the C_1_-cows within the study period (33.4 ± 5.2 kg, mean ± SD), probably resulting from the high genetic potential for milk production of the animals studied within the NCV herd, might have contributed to the inconclusive results.

Avoidance distance was shorter in C_1_-cows than in heifers, which probably should be explained by a good human-animal relationship, particularly owing to caretaker attitudes during milking and to the intensity of management and high number of caretakers [16]. Therefore, our findings ought to be considered for this particular farm/herd, as it should for the finding that in C_1_-cows, age correlated strongly with AD, contrary to the findings of Waiblinger et al. [16] who included several herds. Estimates of avoidance distance at the feeding rack and within the stall correlated moderately, in contrast to Windschnurer et al. [19] who found a stronger correlation (*r* = 0.7–0.9) in a study on 16 commercial dairy farms. Waiblinger et al. [16] found a strong relationship between animals' reactions to humans, particularly avoidance distance inside the stall and the continuity, quality, and quantity of daily contact and handling, and the frequency of friendly interactions with the person milking them (human-animal interactions). Other authors also revealed negative associations between avoidance distances and positive behaviour of milkers on dairy farms and of farmers at bull fattening operations [20, 21]. Accordingly, there is evidence that positive interactions ease handling and milking, increase productivity, and can reduce prevalence of mastitis by promoting adequate milk flow, which has, in additional to improved welfare, an economic impact [4].

The relationship between avoidance distance and oestrous intensity was statistically nonsignificant. No other scientific publication has, to the best of our knowledge, related oestrous intensity with avoidance distance. Possibly, an animal with a good welfare status and, so, properly adapted to its environment, would have a short avoidance distance and, being in a normal physiological state, would have an intense (normal) oestrus. However, avoidance distance cannot be evaluated separately from other welfare criteria. Whay et al. [22] developed a protocol for avoidance distance testing, which would have scored NCV as the first of five categories, with the shortest distances (0.6–1.1 m). Programmes that aim to improve the attitude and behaviour of stockpersons toward dairy cattle can reduce flight distances from humans and increase milk (protein and fat) yields (reviewed by [4, 23]).

Pregnancy respectively calving rates were acceptable for heifers (72%, resp., 64%), but they were rather low among C_1_ cows (37%, resp., 33%). Low fertility is largely attributed to the increase in milk production due to genetic gain, which can explain a part of the low fertility seen in the primiparous cows in the present study [6]. Despite the fact that four heifers—three of them of the SH breed - suffered abortions, the calving results of the present study do not seem to be as poor, particularly for heifers. Whether differences in genetic selection in Scandinavia compared to other regions/countries lay behind the better calving rates for heifers in this herd remains to be determined (see [6]). On the other hand, the low rates depicted by C_1_-cows included in this study deserve attention and further investigation, particularly in relation to AI timing. Attention should be given to individuals with extreme body condition scores, particularly to heifers with high body weight, since they are at risk of becoming problem animals, incurring in repeat breeding (see [6], and references therein).

## 5. Conclusions

Odds of pregnancy-to-term outcome are several times higher when standing oestrus is observed than when animals display only secondary oestrous signs. Monitoring P_4_ levels on-farm could not replace intensive visual detection of oestrus in relation to animal well-being, although avoidance distance was not necessarily related to oestrous intensity.

## Figures and Tables

**Table 1:** Intra- and interassay measurements of relative P_4_ concentrations [low (levels 1-2, <3.0 nM/L), medium (levels 3-4, 3.1–7.5 nM/L) or high (levels 5-6, >7.6 nM/L)), studied in fresh serum, plasma, or milk, or frozen-thawed serum or plasma samples, comparing different sample volumes, at various days (day 0, 7, or 20) of AI, provided by the eProCheck^800^. Number of observations per sample and coefficient of variation (CV) are indicated.

	Intraassay								Interassay		
Sample status	Fresh				Frozen-thawed				Fresh	Frozen-thawed	
Day sampled	d0	d20	d0	d7	d0		d20		d0	d0	d0

Sample type	Serum	Plasma	Milk	Milk	Serum	Plasma	Serum	Plasma	Serum	Serum	plasma

Sample size, **μ**L	15	15	30	20	15	15	15	15	15	15	15

No. of replicates	5	10	10	7	7	7	7	7	7	6	7

Replicates	P_4_-Levels										
1	low	med	low	high	med	high	high	high	low	low	high
2	low	med	med	high	high	high	high	high	low	low	high
3	low	med	low	high	med	high	high	high	low	low	high
4	low	high	med	high	high	high	high	high	med	low	high
5	low	med	low	high	med	high	high	high	low	low	high
6		med	med	high	med	high	high	high	low	low	high
7		med	med	high	high	med	high	high	low	low	high
8		med	low					high			
9		med	med								
10		med	low								

CV (%)	<5.0	15.1	35.1	<5.0	22.0	13.2	<5.0	<5.0	33.1	<5.0	<5.0

**Table 2:** Distribution (frequency and %) of dairy cattle according to oestrous intensity and relative progesterone (P_4_) levels in blood serum on day 0 for all animals, as well as disclosed by category (dairy heifers (H) or first-parity cows (C1 cows)) or breed (Swedish Holstein SH or Swedish Red SR including both H and C1 cows).

	Oestrous intensity								
	Low			Medium			High		
	*Progesterone at d0*								
	*Low*	*Medium*	*High*	*Low*	*Medium*	*High*	*Low*	*Medium*	*High*
All (67)	14/33 (42)^ab^	9/33 (27)^ab^	9/32 (28)^ab^	8/14 (57)^a^	3/14 (21)^ab^	3/14 (21)^ab^	9/20 (45)^a^	8/20 (40)^a^	3/20 (15)^b^
Heifers (37)	7/15 (47)^a^	3/15 (20)^a^	4/14 (29)^a^	2/5 (40)^a^	1/5 (20)^a^	2/5 (40)^a^	6/17 (35)^a^	8/17 (47)^a^	3/17 (18)^a^
C1 cows (30)	7/18 (39)^a^	6/18 (33)^a^	5/18 (28)^a^	6/9 (67)^a^	2/9 (22)^a^	1/9 (11)^a^	3/3 (100)^a^	—	—
SH (36)	8/16 (50)^a^	5/16 (31)^a^	3/16 (19)^a^	4/7 (57)^a^	1/7 (14)^a^	2/7 (29)^a^	7/13 (54)^a^	3/13 (23)^a^	2/12 (17)^a^
SR (31)	6/17 (35)^a^	4/17 (24)^a^	7/17 (41)^a^	4/7 (57)^a^	2/7 (29)^a^	1/7 (14)^a^	2/8 (25)^a^	5/8 (63)^a^	1/8 (13)^a^

^a, b^different superscripts differ significantly, *P* < 0.05 (Fisher's exact test), between P_4_ (all animals) and between animal category (H and C1) and breed (SH and SR), for P_4_ within oestrous intensity level.

**Table 3:** Percentages of pregnancy (animal ratios in parentheses) at day 50 (transrectal manual examination) and of calving, disclosed by relative P_4_ level in blood serum on day 0 and by intensity of oestrus (day 0) for dairy Heifers (*n* = 37) or first parity cows (C1 cows, *n* = 30).

Animal category	P_4_ at day 0	Oestrous intensity at day 0			Pregnancy rates		Calving rates	
		Low	Medium	High				
Heifers	*Low*	71 (5/7)	50 (1/2)	100 (6/6)	80^a^ (12/15)		60 (9/15)	
	*Medium*	67 (2/3)	0 (0/1)	75 (6/8)	67^ab^ (8/12)	72^a^ (26/36)	58 (7/12)	64^a^ (23/36)
	*High*	60 (3/5)	100 (1/1)	67 2/3	67^ab^ (6/9)		78 (7/9)	
C1 cows	*Low*	14 (1/7)	33 (2/6)	67 (2/3)	31^b^ (5/16)		25 (4/16)	
	*Medium*	33 (2/6)	50 (1/2)	—	38^ab^ (3/8)	37^b^ (11/30)	38 (3/8)	33^b^ (10/30)
	*High*	40 (2/5)	100 (1/1)	—	50^ab^ (3/6)		50 (3/6)	
Pregnancy rates		45^a^ (15/33)	46^ab^ (6/13)	80^b^ (16/20)	56 (37/66)			
Calving rates		39^a^ (13/33)	38^a^ (5/13)	75^b^ (15/20)			50 (33/66)	

^a, b^different superscripts differ significantly between animal categories (*P* < 0.01 on PR column and *P* < 0.05 on CR column, PR row and CR row).
